# Prognostic Importance of Fibroblast Growth Factor-23 in Dialysis Patients

**DOI:** 10.1155/2014/602034

**Published:** 2014-09-10

**Authors:** Nilgül Akalin, Yıldız Okuturlar, Özlem Harmankaya, Asuman Gedıkbaşi, Selçuk Sezıklı, Sibel Koçak Yücel

**Affiliations:** ^1^Department of Nephrology, Bakırköy Dr. Sadi Konuk Training and Research Hospital, Istanbul 34147, Turkey; ^2^Department of Internal Medicine, Bakırköy Dr. Sadi Konuk Training and Research Hospital, Istanbul 34147, Turkey; ^3^Department of Biochemistry, Bakırköy Dr. Sadi Konuk Training and Research Hospital, Istanbul 34147, Turkey

## Abstract

*Introduction*. In this study, we aimed to demonstrate the correlation of FGF-23 levels with bone-mineral metabolism, anemia, and the treatment in dialysis patients. *Methods*. Eighty-nine patients with similar age, gender, dialysis duration, and dialysis adequacy who were receiving hemodialysis replacement therapy for at least 6 months were included in the study. Serum iron, iron binding capacity, ferritin, hemoglobin (Hb), hematocrit (Htc), calcium (Ca), phosphorus (P), intact parathormone (iPTH), and FGF-23 levels were studied. In addition, active vitamin D and phosphate binders calcimimetic therapies that patients have received in the last 6 months were recorded. *Results*. It was determined that there was a positive correlation between serum FGF-23 values and PTH values (*P* < 0, 01) and Ca∗P values (*P* < 0, 01). A positive correlation was found between serum FGF-23 values and Ca values at a rate of 24,6% (*P* < 0, 05) and between *P* values at a rate of 59,1% (*P* < 0, 01). A positive correlation was determined between serum FGF-23 values and hemoglobin (Hb) values (*P* < 0, 05) and hematocrit (Htc) values (*P* < 0, 05). In multivariate analysis, no significant correlation was found between serum FGF-23 levels and Hb and Htc. *Conclusion*. The effects of high serum FGF-23 levels on different parameters may be correlated with the development of refractory secondary hyperparathyroidism.

## 1. Introduction

Fibroblast growth factor-23 (FGF-23) is a hormone that has been shown to play a role on the mineralization, vitamin D metabolism, parathyroid gland functions, and phosphate excretion from the kidney. Studies trying to show a correlation between FGF-23 and development of cardiovascular disease and mortality have reported contradictory results [1, 2]. Considering increasing FGF-23 levels with the progression of chronic kidney disease, its effects on the mineral metabolism, and possible relationship with the development of cardiovascular disease, it is thought that FGF-23 might be a prognostic factor [3]. FGF-23, which increases in parallel with the increase of phosphate levels from the early stages of chronic kidney disease, is known to contribute to the development of secondary hyperparathyroidism by leading to suppression of 1,25(OH)2 D levels and increased phosphate excretion [4].

In addition to predicting the prognosis in chronic kidney disease, determination of the correlation between decreased glomerular filtration rate and increased levels of FGF-23 may be an important target, especially in treatment of secondary hyperparathyroidism. In the studies conducted with hemodialysis studies, FGF-23 levels >7500 ng/L have been found to be important in prediction of refractory secondary hyperparathyroidism; however, effects of the secondary hyperparathyroidism treatment on FGF-23 levels is yet to be cleared [5]. In the secondary analysis of the Achieve trial [6], 91 hemodialysis patients were administered low-dose of calcitriol analogues calcimimetic agent cinacalcet and FGF-23 levels were observed to decrease by 9,7% in cinacalcet group.

In Accelerated Mortality on Renal Replacement study [7], phosphate binder therapy was found to be correlated with decreased FGF-23 levels. However, place of vitamin D and calcimimetic in the treatment is unclear.

In this study, we aimed to demonstrate the correlation between FGF-23 and the parameters that have effects on morbidity and mortality in dialysis patients as well as the effect of vitamin D and phosphorus binding calcimimetic on FGF-23.

## 2. Materials and Methods

A total of 89 patients followed up and treated in the Nephrology Polyclinic who were receiving hemodialysis replacement therapy for at least 6 months were included in the study. Patients were divided into two groups according to serum PTH level being below or above 300 pg/mL. Patients were included provided they were similar in terms of age, gender, disease etiology, and dialysis duration and having dialysis adequacy. Patients with chronic and active inflammatory disease (e.g., malignancy, collagen tissue disease, and diabetic foot) and incompliant patients in the treatment were excluded from the study.

Following an overnight fasting, venous blood samples were simultaneously collected from all the patients. Serum LDL-cholesterol, albumin, uric acid, high sensitive C- reactive protein, iron (Fe), iron binding capacity, ferritin, hemoglobin (Hb), hematocrit (Htc), calcium (Ca), phosphorus (P), intact PTH (iPTH), and FGF-23 levels were studied. In addition, Ca∗P ratio was calculated in each patient and averages of active vitamin D and phosphate binders calcimimetic therapies that patients have received in last 6 months were defined. These therapies were recorded. Patients' Kt/V and urea reduction ratio (URR) values were recorded in order to evaluate hemodialysis adequacy.

Total cholesterol, triglycerides, high-density lipoprotein (HDL), and low-density lipoprotein (LDL) cholesterol were analyzed with Architect c16200 Integrated System (Abbott Diagnostics Europe, Wiesbaden, Germany). Plasma levels of high sensitive CRP were measured with Siemens Immulite 2000 device using two-way chemiluminometric immunoassay method.

Serum iPTH levels were determined by Siemens Immulite 2000 immunoassay system (Siemens Healthcare Diagnostics, USA) (normal range < 149 pg/mL). Patients' Kt/V and URR values were recorded in order to evaluate hemodialysis adequacy. Kt/V = −In⁡[(*R* − 0, 008 × *t*)+(4 − 3, 5 × *R*)]ΔBW/BW (*R*: ratio of postdialysis urea to predialysis urea; *t*: dialysis duration; BW: body weight) formula model was used for calculation of Kt/V. [URR = (postdialysis urea/predialysis urea)] formula was used for calculation of URR values. Kt/V > 1, 4 and URR > 70% were considered as dialysis adequacy.

Serum FGF-23 levels were determined and venous blood samples were collected in tubes from the antecubital vein followed by an overnight fasting. The tubes were centrifuged at 2000 g (10 min) to remove the serum. Aliquots of serum samples were stored at −80°C until FGF-23 assaying.

Serum FGF-23 levels were determined using Human FGF-23 ELISA Kit (cat. number EZHFGF-23-32K) purchased from Millipore (USA) following the manufacturer's instructions. Millipore Human FGF-23 ELISA Kit employs the quantitative sandwich enzyme immunoassay technique. Intra-assay and interassay coefficients of variation were 7,2% and 5,3%, respectively. FGF-23 levels were expressed as pg/mL.

This study was approved by the Ethics Committee of Bakırköy Medical Hospital and conducted in accordance with the principles of the Declaration of Helsinki. All participants gave their written informed consent prior to participation in the study.

### 2.1. Statistical Analysis

In this study, statistical analyses were performed using NCSS (Number Cruncher Statistical System) 2007 Statistical Software (Utah, USA) package program.

In evaluation of the data, one-way variance analysis was used in the descriptive statistical methods (mean, standard deviation, median, and interquartile range). Conformity of variables to a normal distribution was provided by applying the logarithmic transformation for FGF-23 and a square root transformation for PTH. Independent *t*-test was used for comparison of two groups and Mann-Whitney *U* test for comparison of the variables with nonnormal distribution. Comparison of the qualitative data was performed using Chi-square test. Lineer regression analysis was carried out in order to define the parameters that affect FGF-23 levels. *P* < 0, 05 values were considered as statistically significant.

## 3. Results

Patients followed up in the Nephrology Polyclinics who were receiving dialysis replacement therapy for at least 6 months were included in the study. Hemodialysis patients were divided into 2 groups as having serum iPTH above or below 300 pg/mL.

Mean age and gender distribution of the patients with serum iPTH levels above and below <300 pg/mL were found with no significant difference between both groups (Table 1).

No significant difference was found between the patient groups that developed etiology of end stage kidney disease (Table 1).

Mean dialysis duration of patients with serum iPTH levels above and below <300 pg/mL were found with no significant difference between both groups (Table 1).

When the patients were divided into 2 groups as having serum iPTH above or below 300 pg/mL, serum levels of FGF-23 were found to be significantly higher in the group with iPTH >300 pg/mL (*P* = 0, 029; Table 1). In the logistic analysis carried out with Ca∗P and PTH a statistical correlation was found (*P* = 0, 0001; Table 1).

Existence of Vitamin D therapy was found to be statistically higher in >300 iPTH group (*n* = 7; 83,90%) than in <300 iPTH group (*n* = 7; 21,2%) (*P* = 0, 0001) (Table 2). Existence of Ca-containing phosphorus binding therapy was found to be statistically higher in >300 iPTH group (*n* = 53; 94,6%) than in <300 iPTH group (*n* = 21; 63,6%) (*P* = 0, 0001) (Table 2).

**Figure 1:**
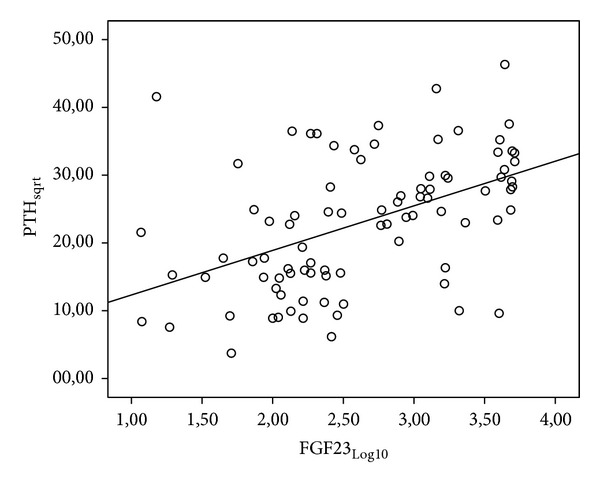
Relationship between FGF-23 and iPTH values.

**Figure 2:**
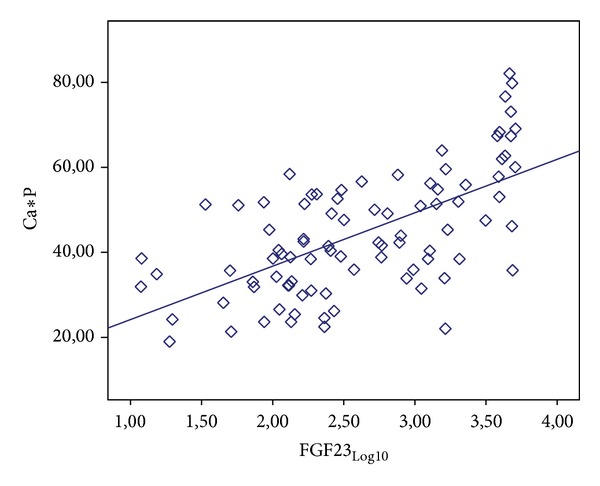
Relationship between FGF-23 and Ca∗P values.

According to the results of univariate analysis, a statistically significant positive correlation was determined between FGF-23 values and iPTH values at a level of 48,0% (*r*: 0,480; *P*: 0,001; *P* < 0, 01) and a statistically significant positive correlation between FGF-23 values and Ca∗P values at a level of 62,3% (*r*: 0,623;  *P*: 0,001; *P* < 0, 01) (Table 3; Figures 1 and 2).

**Figure 3:**
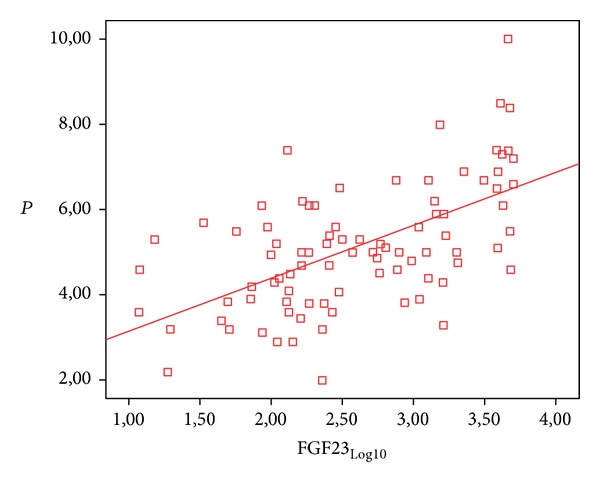
Relationship between FGF-23 and *P* values.

A statistically significant positive correlation was determined between FGF-23 values and Ca values at a level of 24,6% (*r*: 0,246; *P*: 0,021; *P* < 0, 05) and a statistically significant positive correlation between FGF-23 values and *P* values at a level of 59,1% (*r*: 0,591; *P*: 0,001; *P* < 0, 01) (Table 3, Figure 3).

A statistically significant positive correlation was determined between FGF-23 values and Hb values at a level of 24,7% (*r*: 0,247; *P*: 0,020; *P* < 0, 05) and a statistically significant positive correlation between FGF-23 values and Htc values at a level of 24,6% (*r*: 0,246; *P*: 0,020; *P* < 0, 05) (Table 3).

In multivariate analysis, it was observed that the correlation between Hb and Htc values and FGF-23 values becomes insignificant in the model with the effect of the other variables. It was determined that the effects of iPTH and Ca∗*P* values were significant at the level of *P* < 0,01 (Table 3).

## 4. Discussion

Fibroblast growth factor-23 has an important effect on mineral metabolism. Serum FGF-23 levels begin to rise as glomerular filtration rate falls under 90 mL/min/per 1,73 square meters. It was shown that serum FGF-23 levels increased before PTH began to rise in chronic kidney disease [8].

Increased serum FGF-23 levels lead to a decrease in calcitriol production, resulting in secretion of PTH. In our study, we demonstrated a positive correlation between serum FGF-23 and PTH levels. Although the duration of dialysis replacement therapy was the same in all the patients, there were differences between the serum FGF-23 levels of the patients. Today, despite similar duration of disease, etiology, and demographic features, studies conducted in the patients with chronic kidney disease and dialysis patients are trying to explain the differences between the serum FGF-23 levels. Some studies reported that high serum FGF-23 levels had an effect in prediction of resistance to vitamin D therapy and refractory secondary hyperparathyroidism [9]. However, its mechanism of action is yet to be explained. Besides phosphate retention, FGF-23 contributes to progression of parathyroid hyperplasia. Therefore, a correlation has been considered between refractory secondary hyperparathyroidism and FGF-23 levels and importance of FGF-23 in the treatment was begun to be emphasized.

Knowledge about the effects of active vitamin D and calcium-containing phosphorus binding therapies on the serum FGF-23 levels is controversial. In this study, we found serum PTH levels to be elevated in the patients with high serum FGF-23 levels, despite administration of high doses of active vitamin D and calcium-containing phosphorus binding therapies. As it was stated by Gogusev et al. [10], it might be caused by the paradoxical correlation between the serum FGF-23 levels and secondary hyperparathyroidism. According to this opinion, this might be caused by insufficient suppression of parathyroid secretion by the parathyroid gland that remains unresponsive to the calcitriol therapy as well as elevated FGF-23 levels that remain unresponsive in refractory secondary hyperparathyroidism [10, 11]. Although the doses and duration of the therapies were similar in all of the patients in our study, high serum levels in the patients with elevated serum PTH and FGF-23 levels could be insufficient in suppression of PTH secretion and resulted in the development of secondary hyperparathyroidism.

There were some studies indicating that serum FGF-23 levels increased following administration of regular active vitamin D and phosphorus binding treatment compared to pretreatment in hemodialysis patients with severe secondary hyperparathyroidism [12, 13]. However, when we compared the patients with high serum FGF-23 levels, patients with severe secondary hyperparathyroidism, and patients with low and/or normally controlled secondary hyperparathyroidism in this study, we found that the doses of active vitamin D and phosphorus binding treatments were similar. Results obtained from the studies on this issue are controversial. Today, explanation of the FGF23-Klotho axis is becoming increasingly important in order to reveal unknown aspect of the treatment of refractory secondary hyperparathyroidism.

Recent studies have shown that coreceptors Klotho and FGFR1c, which activate FGF-23, decreased in uremic parathyroid hyperplasia, and high serum FGF-23 levels could be explained by Klotho-FGFR1c complexes of secondary hyperparathyroidism [14, 15]. Klotho is produced by the kidneys; therefore, it was seen to decrease in parallel with the loss of the renal functions in chronic kidney disease. Besides mineral metabolism, Klotho FGFR1c-FGF23 axis is considered to be a predictor of progression of kidney disease and to be related to morbidity and mortality. However, this was not fully proven with the current studies.

Secondary hyperparathyroidism contributes to anemia by inhibition of red blood cell production, increasing the fragility and leading to bone marrow fibrosis. Anemia is an important determinant of morbidity and mortality in every stage of kidney disease [16]. Considering the positive correlation between the serum FGF-23 levels and PTH, FGF-23 is believed to contribute to the development of anemia in dialysis patients [16]. However, in our study, we did not determine any correlation between high serum parathormone levels and FGF-23 levels and anemia. This result can be attributed to the regular treatment received by the patient. When the effects of different parameters are excluded, determination of correlation between FGF-23 levels and anemia in the group determined to have high serum FGF-23 levels can support our thought. Correlation between the serum ferritin and FGF-23 levels and effects of the parenteral iron therapy on FGF-23 metabolism are yet to be clarified [17]. In this study, all the patients were receiving parenteral iron therapy at different doses. We found no significant difference between the patients with severe secondary hyperparathyroidism high serum FGF-23 levels and the patients with controlled secondary hyperparathyroidism and low and/or normal serum FGF-23 levels in terms of serum iron and ferritin levels and iron binding capacity.

Gutiérrez et al. [18] found that elevated serum FGF-23 levels were not correlated with mortality in the patient group with low serum phosphorus levels, but they were positively correlated with mortality in the patient group with higher serum phosphorus levels. In another study supporting the aforementioned study, the positive effects of high FGF-23 and phosphorus levels on mortality were attributed to more protein intake of the dialysis patients by the authors [19]. In our study, although albumin values were similar in the patients with low and/or normal or high serum FGF-23 levels, C-reactive protein, P, and Ca∗P values were found to be higher in the patients with high serum FGF-23 levels. Given the different studies, this might indicate increased risk for calcification and inflammation and it might cause negative effects on morbidity. In the studies investigating the effects of FGF-23 on morbidity and mortality, it was reported that FGF-23 might cause peripheral vascular and tissue proliferation by increasing Ca∗P ratio, cellular proliferation, and inflammation and by decreasing calcitriol production and by the toxic effects that we could not explain yet [20, 21].

In this study, we demonstrated that serum FGF-23 levels affected especially the phosphorus metabolism. Besides FGF-23 being a biomarker of phosphate metabolism, we believe that it might also be a biomarker for chronic kidney disease. With a better understanding of the FGF23-Klotho axis, we believe that new approaches might be developed in the treatment of refractory secondary hyperparathyroidism.

## 5. Conclusions

Elevated serum FGF-23 levels prior to determination of iPTH levels in chronic kidney disease and higher FGF-23 levels in patients with refractory secondary hyperparathyroidism assert the importance of FGF23-Klotho axis. We believe that elucidation of FGF23-Klotho will provide a different approach in the treatment of refractory secondary hyperparathyroidism.

## Figures and Tables

**Table 1:** The relationships of demographic features and comparison of serum parathormone levels with fibroblast growth factor-23, calcium, and phosphorus in the dialysis patients.

	PTH < 300 pg/mL	PTH > 300 pg/mL	*P*
Age; mean ± SD	47,94 ± 14,21	46,88 ± 16,57	**0,394**
Gender; *n* (%)			
Male	17 (51,50)	24 (42,90)	**0,506**
Female	16 (48,50)	32 (57,10)	
Dialysis duration (years); mean ± SD	3,03 ± 1,91	4,71 ± 3,03	**0,587**
Etiology; *n* (%)			
Hypertension	14 (42,40)	25 (44,70)	**0,923**
Postrenal KBH	7 (21,30)	7 (12,50)	
Glomerulonephritis	12 (36,40)	23 (41,10)	
Unknown	—	1 (1,80)	
FGF-23_Log 10_ (ng/mL); mean ± SD	159,40 ± 3,66	741,31 ± 4,77	**0,001** ∗∗
Calcium (mg/dL); mean ± SD	8,61 ± 0,99	8,58 ± 0,93	**0,871**
Phosphorus (mg/dL); mean ± SD	4,38 ± 1,07	5,62 ± 1,54	**0,0001**
Ca∗P (mg^2^/dL^2^) mean ± SD	37,77 ± 10,61	48,46 ± 14,75	**0,001**

Data are presented as *n*(%). PTH: parathormone; KBH: chronic renal diseases; FGF-23_  Log 10_: fibroblast growth factor; Ca: calcium; P: phosphorus.

**Statistical significance positive correlation (*P* < 0,01).

**Table 2:** The treatment received according to serum levels of parathormone.

Therapy	PTH < 300 pg/mL	PTH > 300 pg/mL	*P*
Vitamin D			
No	26 (78,80)	9 (16,10)	**0,0001**
Yes	7 (21,20)	47 (83,90)	

Calcium-containing phosphorus binding			
No	12 (36,40)	3 (5,40)	**0,0001**
Yes	21 (63,60)	53 (94,60)	

Data are presented as *n*(%). PTH: parathormone.

**Table 3:** Evaluation of factors affecting FGF23_Log 10_ value.

	Univariate test results		Linear regression analysis results			
	*r*	*P*	*P*	*B*	95% CI	
					Lower	Upper
Constant	—	—	**0,001** ∗∗	1,070	0,673	1,467
PTH_Sqrt_	0,480	**0,001** ∗∗	**0,007** ∗∗	0,018	0,005	0,032
Ca∗P	0,623	**0,001** ∗∗	**0,001** ∗∗	0,025	0,016	0,035
Ca	0,246	**0,021** ∗	—	—	—	—
P	0,591	**0,001** ∗∗	—	—	—	—
Hemoglobin	0,247	**0,020** ∗	**0,965**	−0,009	−0,433	0,414
Hematocrit	0,246	**0,020** ∗	**0,742**	0,004	−0,020	0,028

PTH: parathormone; Ca: calcium.

*Statistical significance positive correlation (*P* < 0,05).

**Statistical significance positive correlation (*P* < 0,01).
